# Treatment Effects of Switching to Faricimab in Eyes with Diabetic Macular Edema Refractory to Aflibercept

**DOI:** 10.3390/medicina60050732

**Published:** 2024-04-28

**Authors:** Tomoaki Tatsumi, Tomomi Kaiho, Takehito Iwase, Gen Miura, Daisuke Shimizu, Tomohiro Niizawa, Yoshihito Ozawa, Miyuki Arai, Toshiyuki Oshitari, Yoko Takatsuna, Takayuki Baba

**Affiliations:** 1 Department of Ophthalmology and Visual Science, Chiba University Graduate School of Medicine, 1-8-1, Inohana, Chuo-ku, Chiba 260-8670, Japan; tomomif24@gmail.com (T.K.); t.iws75@icloud.com (T.I.); gmiura2@chiba-u.jp (G.M.); tomohiro-niizawa@hotmail.co.jp (T.N.); tarii@aol.com (T.O.); yoko_takatsuna@chibah.johas.go.jp (Y.T.); t.baba.oph@faculty.chiba-u.jp (T.B.); 2 Biostatistics Section, Clinical Research Center, Chiba University Hospital, 1-8-1, Inohana, Chuo-ku, Chiba 260-8670, Japan; 3 National Hospital Organization Chiba Medical Center, 4-1-2, Tsubakimori, Chuo-ku, Chiba 260-8606, Japan; 4 Department of Ophthalmology, International University of Health and Welfare School of Medicine, 4-3, Kozunomori, Narita 286-8686, Japan; 5 Chiba Rosai Hospital, 2-16, Tatsumidaihigashi, Ichihara 290-0003, Japan

**Keywords:** diabetic macular edema, angiopoietin-2, vascular endothelial growth factor, faricimab, aflibercept, intravitreal injection, switching

## Abstract

*Background and Objectives*: Faricimab is a vascular endothelial growth factor A and angiopoietin-2 bispecific antibody. It is a novel therapeutic approach distinct from previous anti-vascular endothelial growth factor agents. This study aimed to evaluate the efficacy of switching from aflibercept to faricimab in the treatment of diabetic macular edema (DME) refractory to aflibercept, with a specific focus on the resolution of macular edema. *Materials and Methods*: The medical records of 29 eyes of 21 patients with DME that were refractory to intravitreal injections of aflibercept (IVAs) and who had completed the clinical follow-up of at least four intravitreal injections of faricimab (IVFs) were reviewed. The central retinal thickness (CRT), best-corrected visual acuity (BCVA), and the mean period (weeks) until the next injection were measured after the second-to-last IVA, first-to-last IVA, last IVA, and first to fourth IVFs following the transition to IVF. *Results*: The mean time from the first IVF to the assessment of effectiveness was significantly shorter than the time to the last IVA; however, no significant difference was found in the time from the second, third, and fourth IVFs to the assessment. The mean CRTs after the first and second IVFs were not significantly different from the CRT after the last IVA, but the mean CRT after the third and fourth IVFs was significantly thinner than that after the last IVA (*p* = 0.0025 and *p* = 0.0076, respectively). The mean BCVAs after the third and fourth IVFs significantly improved compared with that after the last IVA (*p* = 0.0050 and *p* = 0.0052, respectively). *Conclusions*: When switching the treatment to IVF for eyes with IVA-resistant DME, better treatment outcomes are achieved if IVF is performed three or more times.

## 1. Introduction

Diabetic macular edema (DME) is the most common cause of reduced visual acuity in patients with non-proliferative diabetic retinopathy [1]. A meta-analysis found that 6.81% of the 22,896 patients surveyed had DME [2]. Intravitreal injections of anti-vascular endothelial growth factor (anti-VEGF) agents, such as bevacizumab, ranibizumab, and aflibercept, have emerged as globally recognized standard therapies for DME [3,4].

Although anti-VEGF treatments have demonstrated efficacy in resolving DME, the recurrence rate of DME remains high [5]. Thus, multiple anti-VEGF injections are required to maintain ME resolution. Some patients are resistant to anti-VEGF treatment, and a certain number of patients exhibit resistance even after receiving consecutive monthly injections; the incidence of DME persisting for 24 weeks is approximately 30%, even with at least four regular injections of aflibercept [6]. Undoubtedly, VEGF is a key factor in the development of DME; however, inflammatory factors such as intercellular adhesion molecule 1 (ICAM-1), interleukin-6 (IL-6), and monocyte chemotactic protein-1 (MCP-1) have demonstrated correlations [7]. Moreover, inflammatory factors like tumor necrosis factor-α (TNF-α), IL-1α, IL-1β, IL-8, IL-10, and placental growth factor (PlGF) have been identified as contributors to the inflammatory processes underlying the development of DME [8].

Therefore, treatments targeting angiopoietin (Ang) and tyrosine kinase with immunoglobulin-like and epidermal growth factor homology domain (Tie) signaling pathways have been investigated [9,10].

The outcomes of treatment with faricimab (Roche/Genentech, Basel, Switzerland), a bispecific antibody for Ang-2 and VEGF-A, appeared to be in line with the findings of these studies. Prior to the introduction of faricimab, no DME treatments that could inhibit Ang-2 were available, and this agent was expected to exert therapeutic effects through a different mechanism. However, a few studies have demonstrated the effects of faricimab in eyes with DME refractory to intravitreal aflibercept (Bayer Consumer Care AG, Basel, Switzerland) (IVA) treatment [11,12,13]. This study aimed to evaluate the efficacy of switching aflibercept to faricimab in treating eyes with refractory DME following IVA injections.

## 2. Materials and Methods

This study included patients treated with existing anti-VEGF agents at Chiba University Hospital, predominantly under the treat-and-extend (TAE) regimen, whose macular edema did not resolve or repeatedly recurred following this treatment. The medical records of 29 eyes of 21 patients whose treatment was switched to faricimab between September 2022 and March 2023 and who received four or more injections without changing the treatment regimen were examined. All patients received at least four IVAs before switching to intravitreal faricimab (IVF) injections.

The central retinal thickness (CRT), best-corrected visual acuity (BCVA), and interval between injections were assessed. The intervals between treatments were determined based on the results of the previous treatments.

**Figure 1:**
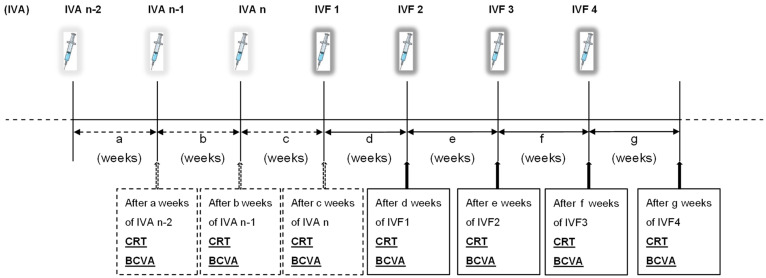
Overview of this study protocol. The treatment was switched to intravitreal injection of faricimab (IVF) for patients with diabetic macular edema (DME) who were refractory even after receiving multiple intravitreal injections of aflibercept (IVAs) under the treat-and-extend (TAE) regimen. The central retinal thickness (CRT), best-corrected visual acuity (BCVA), and injection-to-injection intervals were measured after three IVAs before switching (IVA n-2, IVA n-1, and IVA n) and four IVFs (IVF1, IVF2, IVF3, and IVF4) post-switch.

In this study, we evaluated the parameters after three IVAs before switching (IVA n-2, IVA n-1, and IVA n) as well as the measured values after four IVFs following the transition (IVF1, IVF2, IVF3, and IVF4) (Figure 1). Furthermore, we evaluated the influence of certain factors such as the duration before switching to another agent, number of injections, forms of edema, and responsiveness to anti-VEGF treatment (ineffectiveness or relapse) on the results. As this was a retrospective study that relied on medical records, it included patients who received retreatment prior to fulfilling the established criteria and cases where a strict adherence to retreatment intervals was not followed.

### 2.1. Inclusion Criteria

Patients who had undergone IVA treatment for DME, yet experienced persistent or recurrent macular edema, and met the following criteria were included in the study:

1. Even after undergoing IVA treatment four times or more, the CRT remained at 370 μm or higher after each IVA session.
2. Despite undergoing IVA treatment eight times or more, the CRT initially reduced to <350 μm following IVA treatment but eventually increased to >400 μm or worsened by more than 100 μm within 12 weeks.

### 2.2. Exclusion Criteria

1. Patients with active proliferative diabetic retinopathy requiring vitrectomy;
2. Patients with thickened posterior hyaloid membrane and epiretinal membrane of the macula;
3. Patient receiving hemodialysis treatment;
4. Patients with poorly controlled diabetes and hypertension or those who started treatment for diabetes within 12 months;
5. Patients with cataracts that affected visual acuity;
6. Patients with other retinal diseases such as age-related macular degeneration or retinal vein occlusion;
7. Patients who underwent vitrectomy within 24 months before the second-to-last IVA (IVA n-2);
8. Patients who underwent retinal photocoagulation within 24 months before switching to IVF.

### 2.3. Determination of Retreatment Intervals

The treatment intervals for anti-VEGF agents were determined based on the time that patient consent was obtained and the judgment of each physician:

1. The minimum treatment interval was 4 weeks.
2. If the CRT increased by 50 μm or more from that of the previous visit, the interval was shortened by two weeks.
3. If CRT decreased to <325 µm [10], the intervals were extended by 2 weeks. However, even if the CRT criterion was met, the interval was maintained if retinal edema occurred within 500 µm from the fovea or if exacerbation of hard exudate near the fovea was observed.

CRT was measured in images obtained using spectral-domain optical coherence tomography (Heidelberg Engineering, Heidelberg, Germany). The macular thickness map program was used to obtain the CRT data of all patients.

BCVA was measured using a Landolt chart at each visit. The same protocol was used to measure BCVA in all patients. All BCVA measurements and OCT images were obtained by one of the seven orthoptists.

All patients received intravitreal injections of aflibercept (Bayer Consumer Care AG, Basel, Switzerland) (IVAs) or faricimab (Roche/Genentech, Basel, Switzerland) (IVFs) using a standard method without complications. For IVA, 2 mg of aflibercept was injected; for IVF, 6.0 mg of faricimab was injected intravitreally. Topical antibiotics were administered 3 days before and after treatment.

The clinical data and demographics of the patients before switching from IVA to IVF are presented in Table 1.

### 2.4. Statistical Analyses

Statistical analyses were performed using JMP Pro version 16 software (SAS Institute, Cary, NC, USA). The patient demographics characteristics and the baseline characteristics are presented as means with standard deviations (mean ± SD) and ranges (min and max) for continuous variables and frequency and percentage for categorical variables. The longitudinal data analysis of effects after each injection period was analyzed using a mixed-effects model, with means and standard errors shown (mean ± SEM). CRT, BCVA, and treatment intervals in 29 eyes of 21 patients were analyzed using a mixed-effects model with the number of injections as a fixed effect and the patients and the right or left eye branched from the patients as a random effect. Student’s *t*-tests were used to compare the treatment effects on different types of DME. Pearson’s product–moment correlation coefficient was used to analyze the relationship between the number of IVAs before switching to IVFs and the change in CRT after switching. A *p* value of <0.05 was considered significant. For statistical analyses, the decimal BCVA measured using a Landolt chart was converted to logMAR units.

## 3. Results

### 3.1. CRT, BCVA, and Period until the Evaluation of Effects after Each Injection

**Figure 2:**
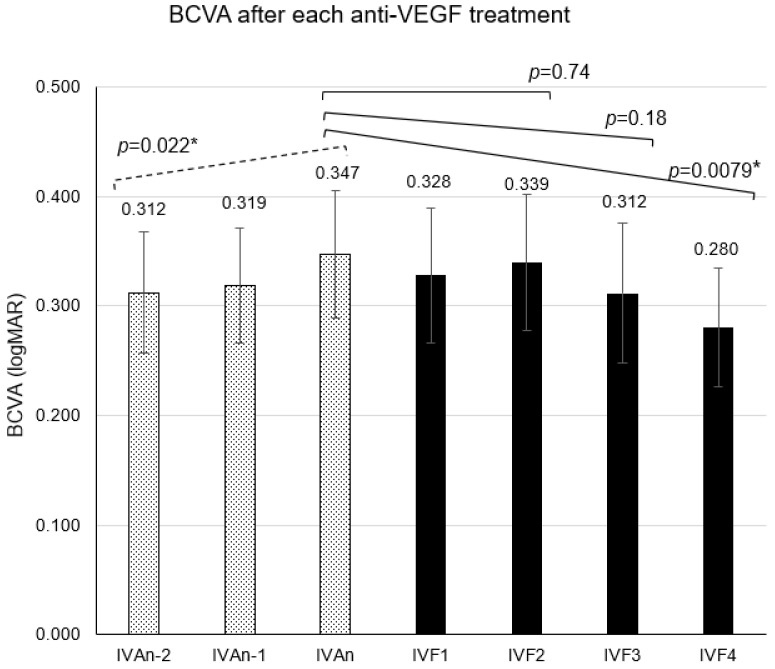
The mean best-corrected visual acuity (BCVA) after the n-2nd intravitreal injection of aflibercept (IVAn-2) to the 4th intravitreal injection of faricimab (IVF4). The effect after each injection was measured at the time of the subsequent injection. The mean BCVA values were 0.312 ± 0.055 after IVAn-2, 0.319 ± 0.052 after IVAn-1, 0.347 ± 0.058 after IVAn, 0.328 ± 0.062 after IVF1, 0.339 ± 0.062 after IVF2, 0.312 ± 0.064 after IVF3, and 0.280 ± 0.054 after IVF4. Error bars represent standard error of the mean (SEM). *: This value means *p* < 0.05 in mixed-effects model.

**Figure 3:**
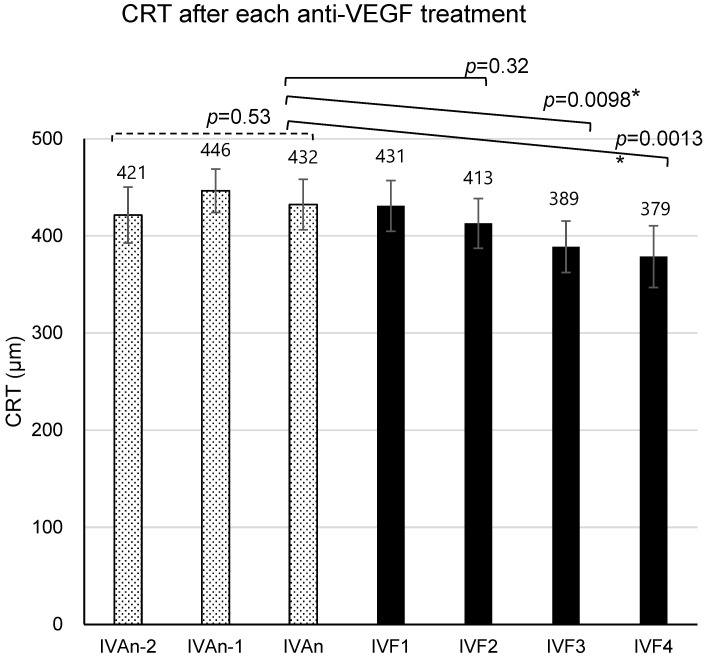
The mean central retinal thickness (CRT) after the n-2nd intravitreal injection of aflibercept (IVAn-2) to the 4th intravitreal injection of faricimab (IVF4). The effect after each injection is measured at the time of the subsequent injection. The mean CRT values were 421 ± 29 µm after IVAn-2, 446 ± 22 µm after IVAn-1, 432 ± 26 µm after IVAn, 431 ± 26 µm after IVF1, 413 ± 26 µm after IVF2, 389 ± 27 µm after IVF3, and 379 ± 32 µm after IVF4. Error bars represent standard error of the mean (SEM). *: This value means *p* < 0.05 in mixed-effects model.

**Figure 4:**
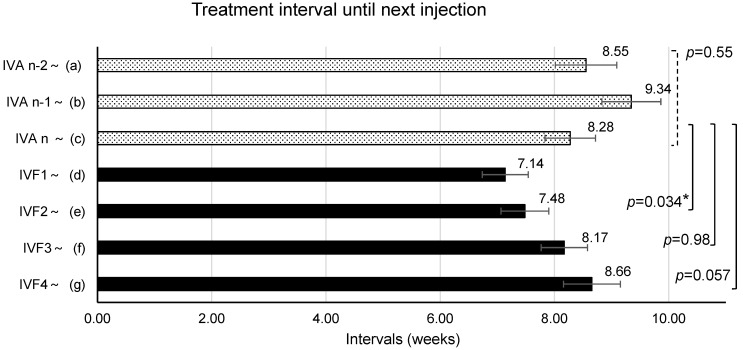
The mean interval until the next injection (periods to determine the effectiveness of previous intravitreal injection) after the n-2nd intravitreal injection of aflibercept (IVAn-2) to the 4th intravitreal injection of faricimab (IVF4). The mean intervals were 8.55 ± 0.54 weeks after IVAn-2, 9.34 ± 0.52 weeks after IVAn-1, 8.28 ± 0.44 weeks after IVAn, 7.14 ± 0.40 weeks after IVF1, 7.48 ± 0.42 weeks after IVF2, 8.17 ± 0.41 weeks after IVF3, and 8.66 ± 0.50 weeks after IVF4. Error bars represent standard error of the mean (SEM). *: This value means *p* < 0.05 in mixed-effects model.

The CRT, BCVA (logMAR), and periods (mean ± standard error of the means) until the evaluation of the effects after the second-to-last IVA (IVAn-2) were 421 ± 29 µm, 0.312 ± 0.055, and 8.55 ± 0.54 weeks, respectively. After the first-to-last IVA (IVAn-1), the values obtained were 446 ± 22 µm, 0.319 ± 0.052, and 9.34 ± 0.52 weeks, respectively. After the last IVA (IVAn), first IVF (IVF1), second IVF (IVF2), third IVF (IVF3), and fourth IVF (IVF4), the values obtained were 432 ± 26 µm, 0.347 ± 0.058, and 8.28 ± 0.44 weeks; 431 ± 26 µm, 0.328 ± 0.062, and 7.14 ± 0.40 weeks; 413 ± 26 µm, 0.339 ± 0.062, and 7.48 ± 0.42 weeks; 389 ± 27 µm, 0.312 ± 0.064, and 8.17 ± 0.41 weeks; and 379 ± 32 µm, 0.280 ± 0.054, and 8.66 ± 0.50 weeks, respectively (Figure 2, Figure 3 and Figure 4).

When the clinical course of CRT from IVAn-2 to IVAn during IVA treatment was analyzed using a mixed-effects model, no significant improvement or deterioration was found (*p* = 0.53). On the other hand, analysis of the clinical course of CRT from IVAn to IVF4 after switching to IVF using a mixed-effects model showed a significant improvement (*p* = 0.0013). Significant improvements were also seen in the analysis of CRT from IVAn to IVF3 (*p* = 0.0098). However, analysis of CRT from IVAn to IVF2 showed no significant changes (*p* = 0.32).

When the clinical course of BCVA (LogMAR) from IVAn-2 to IVAn during IVA treatment was analyzed, a significant trend of worsening was found (*p* = 0.022). On the other hand, analysis from IVAn to IVF4 after switching to IVF showed a significant BCVA improvement (*p* = 0.0079). Analysis of BCVA from IVAn to IVF3 and from IVAn to IVF2 showed no significant changes (*p* = 0.18, 0.74). 

When the interval from IVAn-2 to IVAn during IVA treatment was analyzed, no significant changes were found (*p* = 0.55). Analysis of the treatment interval from IVAn to IVF2 after switching to IVF showed a significant shortening (*p* = 0.034), but no significant change was observed from IVAn to IVF3 (*p* = 0.98) and IVAn to IVF4 (*p* = 0.057).

During the observation period, none of the patients required additional treatment due to adverse events such as the elevation of intraocular pressure, intraocular inflammation, occlusion of retinal vessels, and infection.

### 3.2. CRT Change Rate after Switching to Faricimab

**Figure 5:**
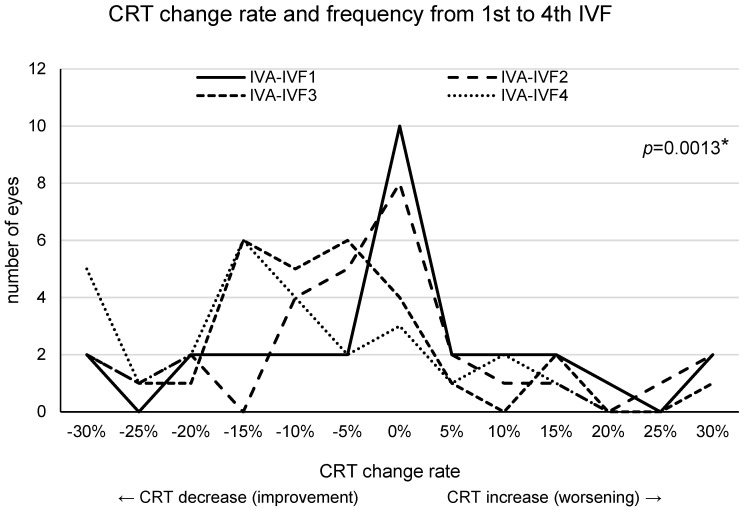
This figure shows the change rate of the central retinal thickness (CRT) after IVF1 to IVF4 compared with that after the final intravitreal injection of aflibercept (IVAn). The vertical axis plots the number of eyes, with the number of eyes at 0% representing the number of eyes with a −5% to 5% change in CRT compared to CRT after the final IVA. Only minimal change was observed in the CRT after the first intravitreal injection of faricimab (IVF1) compared with that after the IVAn. Conversely, in many eyes, the CRT after IVF4 decreased compared with that after IVAn. CRT decreased gradually but significantly over the course of four IVF treatments (*p* = 0.0013, mixed-effects model). *: This value means *p* < 0.05 in mixed-effects model.

The CRT change rate after switching to the first to fourth faricimab regimens is shown in Figure 5. The majority of patients showed no difference in CRT change rates after the first switch to IVF, with only a few patients experiencing worsening CRTs post-switch. However, when IVF was repeated more than twice, the proportion of patients showing improvements in CRTs increased, accompanied by an increase in the improvement rate.

### 3.3. Treatment Effects for Different Types of Macular Edema

**Figure 6:**
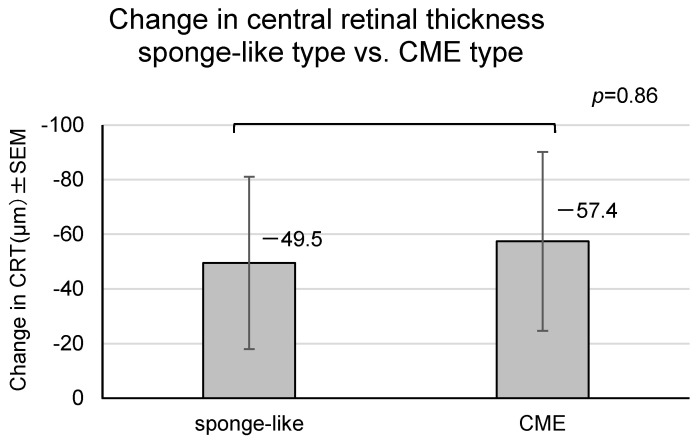
This figure shows the change in the central retinal thickness (CRT) of the sponge-like type and cystoid macular edema (CME) type. The changes in CRT from IVAn to IVF4 are compared. Error bars represent standard error of the mean (SEM). *: This value means *p* < 0.05 in Student’s *t*-test.

Upon transitioning from IVA to IVF or within 12 weeks after the last recurrence of macular edema, macular edema was divided into three types [14]: sponge-like (*n* = 14), cystoid macular edema (CME) (*n* = 15), and serous retinal detachment (*n* = 0). The variations in CRT from IVAn to IVF4 were compared between the sponge-like and CME types. The change in CRT of the sponge-like type was −49.5 ± 31.5 µm, showing no significant difference from that of the CME type (−57.4 ± 24.0 µm) (Figure 6, *p* = 0.86, Student’s *t*-tests). These results indicated that the type of macular edema was not correlated with the effect of IVF treatment on macular edema after switching from IVA.

### 3.4. Relationship between the Number of IVAs before Switching to IVF and the Change in CRT after Switching

**Figure 7:**
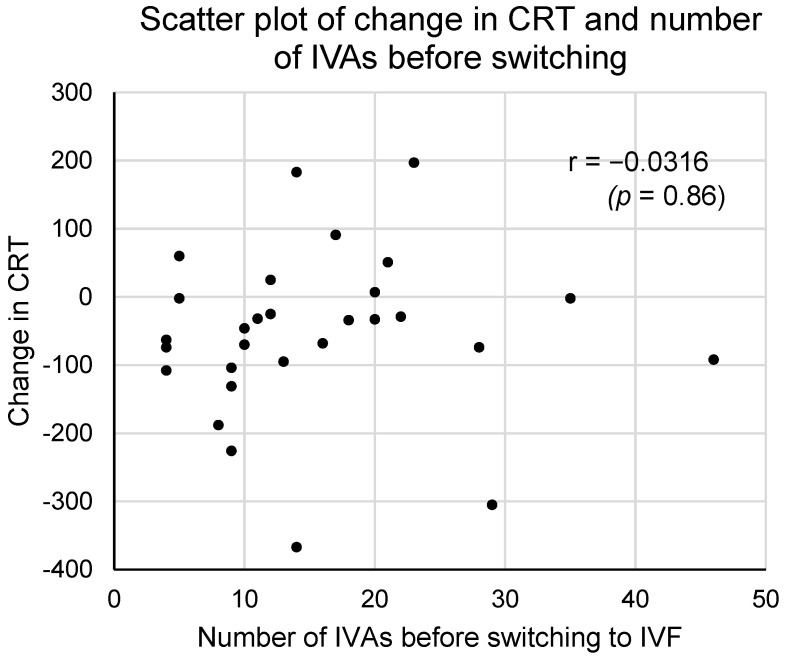
This figure is a scatterplot showing the relationship between the change in central retinal thickness (CRT) from IVAn to IVF4 and the number of IVAs before switching to IVF. There was no significant correlation between these two datasets (Pearson product–moment correlation coefficient: −0.0316, *p* = 0.86).

Before switching to IVF, IVAs performed 15.4 ± 9.7 times. The correlation between the time of IVA injection and the change in CRT after switching to IVF was shown in Figure 7. It was not significant (Pearson product–moment correlation coefficient: r = −0.0316, *p* = 0.86). These results suggest that the number of IVAs before switching to IVF is not related to the IVF effect on DME after switching.

### 3.5. Cases with Limited Response and Cases with Recurrence

**Figure 8:**
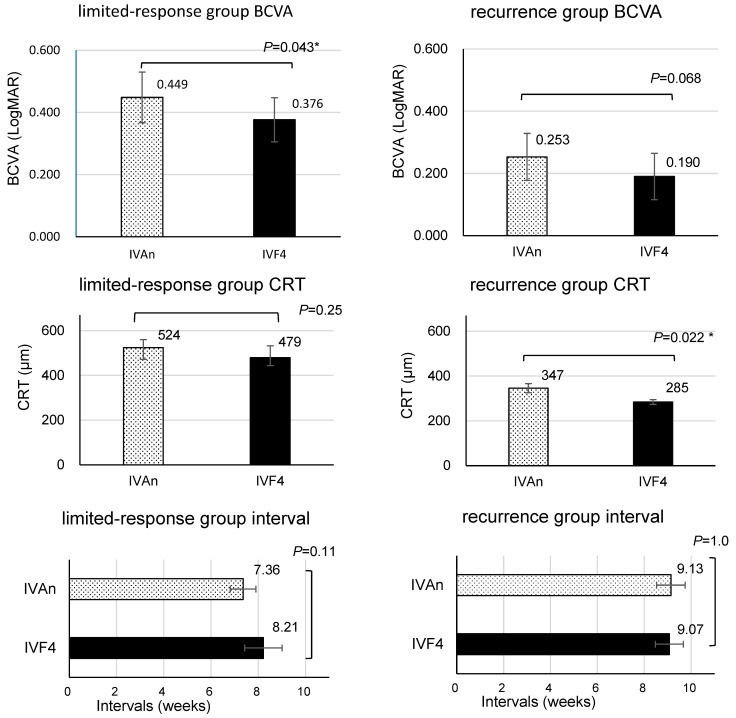
The refractory patients included in this study were categorized into two groups: the limited-response group, comprising individuals with a restricted response to IVA (14 eyes), and the recurrence group, consisting of individuals whose DME improved but recurred in a short timeframe (15 eyes). A comparative analysis of BCVA, CRT, and intervals was conducted for both groups after IVAn and after IVF4 using a mixed model. Error bars represent standard error of the mean (SEM). *: This value means *p* < 0.05 in mixed-effects model.

In this study, refractory patients were classified into two groups: the limited-response group and the recurrence group. The limited-response group comprised patients who experienced limited aflibercept treatment effects and insufficient improvement in edema. The recurrence group included patients whose edema initially improved but eventually recurred within a short period. The former group was defined based on inclusion criterion (1) described in the Materials and Methods section, while the latter group was enrolled in the study based on criterion (2). The former was referred to as the limited-response group, while the latter was referred to as the recurrence group. Fourteen eyes from ten patients in the limited-response group and fifteen eyes from thirteen patients in the recurrence group were evaluated. Figure 8 shows the BCVAs, CRTs, and intervals after IVAn and IVF4 in both groups.

### 3.6. Switching Effect for Cases with Limited Response to IVA and Cases with Short-Term Recurrence

Although no significant differences were observed owing to the small number of patients, both groups exhibited a comparable trend of improvement in BCVAs and CRTs. Although no significant difference was found in the limited-response group, the interval was slightly longer with IVF4, with both BCVA and CRT showing a tendency to improve. In the recurrence group, CRT significantly decreased, while BCVA tended to improve. These results indicate that after IVF4, only a few of the patients experienced edema recurrence before the next IVF. The period until recurrence was also extended. Six out of fifteen eyes showed a worsening of the CRT (≥50 μm from the lowest CRT value) during IVA treatment before switching, but none of the eyes experienced this event after IVF4. Although both groups showed improvement after switching to IVF, it should be noted that baseline CRT and BCVA were better in the recurrence group.

## 4. Discussion

A recent Japanese survey indicated that a majority of ophthalmologists prefer intravitreal injections of anti-VEGF agents as first-line therapy [15]. The survey also showed that the most common approach during the loading phase was a single initial injection (51.4%), followed by three consecutive monthly injections (26.1%); the pro re nata (PRN) regimen was preferred (75.0%) during the maintenance phase after a successful loading phase [15]. Another recent real-world study conducted in Japan reported an annual increase in the number of anti-VEGF agent injections per case [16].

In the present study, patients with prolonged treatment periods received a single injection during the loading phase and a PRN regimen during the maintenance phase (1 + PRN). However, if treatment was initiated recently, three consecutive monthly injections were administered during the loading phase. Given that all patients in this study were resistant to IVA, they were treated with a treat-and-extend regimen before switching to IVF.

As all patients in this study were refractory to IVA, the IVA treatment was switched to IVF with the hope of achieving further improvement. However, macular edema may worsen when switching treatments. Therefore, the interval from IVAn to IVF1 (7.14 ± 2.16 weeks) was shorter than the interval from the previous aflibercept (8.28 ± 2.38 weeks) (Figure 4). In fact, two out of the twenty-nine eyes experienced a worsening of macular edema (CRT worsened by ≥30%). However, the majority of the remaining 27 eyes experienced ≤20% worsening of macular edema, remained stable, or showed improvement. The binding affinity of faricimab to VEGF-A is similar to that of ranibizumab [17], and that of aflibercept is higher than that of ranibizumab [18,19]. Therefore, differences may exist between the anti-VEGF effects of the two agents. In the two cases in which CRT worsened after the first IVF, the anti-Ang-2 effect of faricimab had no effect on refractory macular edema with excessive pericyte damage, with the primary influence being the anti-VEGF-A effect. However, the binding affinity for VEGF-A may not be directly linked to the improvement of macular edema, and responsiveness to the drug may differ among patients. Although CRT increased in two patients, it decreased by more than 30% in another two patients, while it remained unchanged in most patients.

Both CRT and BCVA gradually improved with each IVF treatment, with no change in the first and second treatments but a significant decrease in the third and fourth treatments. This observed trend may be attributed to the anti-Ang-2 effect of faricimab, which is not exerted by aflibercept. The delayed onset of this effect could be due to the time required for pericytes to be repaired with faricimab. However, the precise duration for the anti-Ang-2 effects to reduce macular edema remains unclear. Further studies are required to clarify the time lag in the effect of IVF, switched from IVA, on refractory macular edema.

In this study, three response variables (CRT, BCVA, and treatment interval) were evaluated. Since CRT and BCVA are correlated and are measured under the TAE regimen, the treatment interval is determined by CRT. Therefore, it is important to note that these three variables are not independent but depend on each other. In this study, during three IVA treatments (IVAn-2, IVAn-1, and IVAn), both CRT and BCVA did not improve or worsened, and the treatment interval did not change. We considered that switching to IVF would be effective if CRT or both CRT and BCVA improved after switching to IVF, and the treatment interval remained unchanged or was extended.

A recent study indicated a possible risk of sustained IOP elevation of approximately 7% after repeated anti-VEGF injections in patients with DME without a history of glaucoma [20]. In this study, 1.4% of patients required treatment. Although a simple comparison could not be made, the percentage was lower than that with topical steroid treatments.

The risk of IOP elevation after frequent anti-VEGF injections has been reported to be approximately 7% in AMD [21]. The mean duration until elevation from baseline was 50.6 ± 26.5 months, indicating a gradual increase in IOP after an extended period. Therefore, it is important to establish a treatment method for refractory cases that does not rely on steroids to broaden the available options. Highly functional or value-added anti-VEGF drugs will serve as alternative treatment options for refractory cases, and faricimab, with added anti-Ang-2 activity, is expected to be a promising treatment option.

However, only a few studies have reported the effects of faricimab on DME in clinical practice. Kusuhara et al. reported that the course of both CRT and BCVA did not differ between treatment-naïve and switch groups [11]. In Kusuhara’s study, although the follow-up period was 6 months and the treatment protocol was intravitreal injection as needed (pro re nata: PRN), the switch group, previously treated with other anti-VEGF agents (mostly aflibercept), exhibited comparable improvement to the treatment-naïve group. This suggests that the anti-Ang-2 effect of faricimab may be responsible for this improvement.

In patients with DME that is refractory to other anti-VEGF agents (aflibercept and ranibizumab), as observed in this study, Ohara et al. reported that switching to IVF may extend the treatment interval [12]. Similarly, Rush et al. reported the effectiveness of switching to IVF using the TAE protocol in patients with DME refractory to IVA [13]. In their analysis of 51 patients with 51 eyes, they reported that a longer treatment interval could be achieved, with both BCVA and CRT significantly improving 12 months after the switch. The present study reported similar results; however, significant improvements in CRT and BCVA were observed after three or more injections. Although the treatment interval was not extended until the fourth IVF cycle after the switch, a gradual extension was observed.

Initiating anti-VEGF treatment for DME with three to five initial monthly doses is effective [3,22,23]. This protocol is increasingly adopted in clinical practice [15]. Existing studies suggest that administering three initial monthly doses is more effective than a single dose [24]. Large-scale clinical trials of faricimab are underway using a protocol with four initial monthly doses; therefore, this protocol is recommended for DME treatment [10]. This protocol may be more effective when switching to faricimab therapy. Thus, it may have been possible to reduce the number of patients experiencing worsening after switching to faricimab. However, obtaining consent for the protocol with four monthly injections can be challenging, especially if patients have been previously treated with a TAE regimen using aflibercept. The advantages of continuing the TAE protocol in this study include the ease of comparing effects before and after switching and a simplified process for obtaining patient consent.

Factors that influence the effectiveness of anti-VEGF therapy include vitrectomy, photocoagulation (PC), pan-retinal photocoagulation (PRP), sub-tenon triamcinolone acetonide injection (STTA), and intravitreal triamcinolone acetonide injection (IVTA). In particular, it has been reported that a history of STTA may be less likely to be associated with a longer recurrence interval after switching to IVF [12]. Eyes with an interval of 24 months or less between the final vitrectomy/PC/PRP and the switch to IVF were not included in this study. STTA/IVTA was performed in 11 of the 29 eyes (Table 1). In only one eye, the interval between final STTA/IVTA and switching to IVF was 13 months, while in the other 10 eyes it was more than 24 months. Although the influence may not be large, caution is required.

The average number of IVAs performed before switching to IVF was 15.4 ± 9.7 (mean ± standard deviation), with a range of 4 to 46 and a median of 13 (Table 1). In this study, the number of IVAs before switching varied considerably, ranging from 4 to 46, which may have influenced the effectiveness after switching to IVF. A smaller number of IVAs might suggest that the observed effect is not due to the difference between the effects of IVA and IVF but rather because a limited amount of IVA treatment is not sufficiently effective, and the effect becomes apparent after switching to IVF. However, no significant correlation was observed between the number of IVAs before switching to IVF and the change in CRT after switching to IVF (from CRT after IVF4 to CRT after IVAn) (Figure 7, Pearson product–moment correlation coefficient: −0.0316). The efficacy of switching to IVF was not significantly influenced by the number of IVAs administered prior to switching. It cannot be conclusively stated that IVF was solely effective due to the smaller number of IVAs.

In this study, to evaluate the effectiveness of switching to IVF, we enrolled two different types of cases: cases with limited response and cases with short-term recurrence. This may make it difficult to analyze the three variables of CRT, BCVA, and the treatment interval. It would be easier to interpret the effects if the research was conducted with the same treatment intervals for each type. Additionally, in this study, both groups improved after switching, and the recurrence group showed somewhat better improvement. However, it should be noted that the baseline CRT and BCVA were better in the recurrence group.

This study has some limitations. First, it was a retrospective study. Second, the sample size was small. Third, as real-world clinical data, protocols such as the retreatment criteria and treatment intervals were not strictly enforced. Thus, further large-scale prospective studies are required to evaluate the efficacy and safety of switching to IVF for refractory DME.

## 5. Conclusions

In conclusion, switching to IVF appears to be a beneficial approach for managing refractory DME in eyes that have received multiple IVA treatments. However, this option may not be effective after only one injection post-switch; certain patients might even experience a worsening of their condition. However, a positive effect may become evident with continued injections, specifically, three or more times.

## Figures and Tables

**Table 1:** Baseline (just before switching to IVF) characteristics of patients or eyes immediately before switching to IVF.

Sex Male or female, patients	Male, *n* (%)	12	(57.1%)
	Female, *n* (%)	9	(42.9%)
Age, years	Mean ± SD, [range]	59.8 ± 12.0, [36–77]	
HbA1c, %	Mean ± SD, [range]	6.83 ± 1.14, [5.6–10.8]	
Hypertension, patients	*n* (%)	12	(52.1%)
Dyslipidemia, patients	*n* (%)	9	(42.9%)
Previous history of PC, eyes	PRP, *n* (%)	17	(58.6%)
	Focal PC, *n* (%)	1	(3.4%)
	None, *n* (%)	11	(37.9%)
Previous history of PPV, eyes	*n* (%)	5	(17.2%)
Number of anti-VEGF injections before switching to IVF	Mean ± SD, [range]	15.4 ± 9.7, [4–46]	
Previous history of STTA/IVTA, eyes	*n* (%)	11	(37.9%)
Interval between final STTA/IVTA and switching to IVF, months	Mean ± SD, [range]	30.3 ± 9.1, [13–45]	
Number of STTA before switching to IVF	Mean ± SD, [range]	0.55 ± 0.77, [0–2]	
Number of IVTA before switching to IVF	Mean ± SD, [range]	0.24 ± 0.77, [0–4]	
Lens status, eyes	phakia, *n* (%)	19	(65.5%)
	pseudophakia, *n* (%)	10	(34.5%)
CRT, μm	Mean ± SD, [range]	432 ± 140, [251–878]	
BCVA (logMAR)	Mean ± SD, [range]	0.347 ± 0.314, [−0.079–1.097]	
Periods from the last IVA, weeks	Mean ± SD, [range]	8.28 ± 2.38, [5–15]	

SD, standard deviation; HbA1c, glycated hemoglobin; PC, photocoagulation; PRP, pan-retinal photocoagulation; PPV, pars plana vitrectomy; VEGF: vascular endothelial growth factor; IVF, intravitreal injections of faricimab; STTA, sub-tenon triamcinolone acetonide injection; IVTA, intravitreal triamcinolone acetonide injection; CRT, central retinal thickness; BCVA, best-corrected visual acuity; IVA, intravitreal injections of aflibercept.

## Data Availability

The data used to support the findings of this study are available upon request from the corresponding author.
